# Development and Validation of a Predictive Risk Score for Blood Transfusion in Patients Undergoing Curative‐Intent Surgery for Intrahepatic Cholangiocarcinoma

**DOI:** 10.1002/jso.27903

**Published:** 2024-09-16

**Authors:** Giovanni Catalano, Laura Alaimo, Yutaka Endo, Odysseas P. Chatzipanagiotou, Andrea Ruzzenente, Luca Aldrighetti, Matthew Weiss, Todd W. Bauer, Sorin Alexandrescu, George A. Poultsides, Shishir K. Maithel, Hugo P. Marques, Guillaume Martel, Carlo Pulitano, Feng Shen, François Cauchy, Bas G. Koerkamp, Itaru Endo, Minoru Kitago, Timothy M. Pawlik

**Affiliations:** ^1^ Department of Surgery The Ohio State University Wexner Medical Center and James Comprehensive Cancer Center Columbus Ohio USA; ^2^ Department of Surgery University of Verona Verona Italy; ^3^ Department of Surgery Ospedale San Raffaele Milan Italy; ^4^ Department of Surgery Johns Hopkins Hospital Baltimore Maryland USA; ^5^ Department of Surgery University of Virginia Charlottesville Virginia USA; ^6^ Department of Surgery Fundeni Clinical Institute Bucharest Romania; ^7^ Department of Surgery Stanford University Stanford California USA; ^8^ Department of Surgery Emory University Atlanta Georgia USA; ^9^ Department of Surgery Curry Cabral Hospital Lisbon Portugal; ^10^ Department of Surgery University of Ottawa Ottawa Ontario Canada; ^11^ Department of Surgery, Royal Prince Alfred Hospital University of Sydney Sydney New South Wales Australia; ^12^ Department of Surgery Eastern Hepatobiliary Surgery Hospital Shanghai China; ^13^ Department of Hepatobiliopancreatic Surgery and Liver Transplantation, AP‐HP Beaujon Hospital Clichy France; ^14^ Department of Surgery Erasmus University Medical Centre Rotterdam The Netherlands; ^15^ Department of Gastroenterological Surgery Yokohama City University School of Medicine Yokohama Japan; ^16^ Department of Surgery Keio University Tokyo Japan

**Keywords:** anemia, blood transfusion, cholangiocarcinoma, liver resection

## Abstract

**Background and Objectives:**

Among patients undergoing liver resection for intrahepatic cholangiocarcinoma (ICC), perioperative bleeding requiring blood transfusion is a common complication, yet preoperative identification of patients at risk for transfusion remains challenging. The objective of this study was to develop a preoperative risk score for blood transfusion requirement during surgery for ICC.

**Methods:**

Patients undergoing curative‐intent liver surgery for ICC (1990–2020) were identified from a multi‐institutional database. A predictive model was developed and validated. An easy‐to‐use risk calculator was made available online.

**Results:**

Among 1420 patients, 300 (21.1%) received an intraoperative transfusion. Independent predictors of transfusion included severe preoperative anemia (OR = 1.65, 95% CI 1.10–2.47), T2 category or higher (OR = 2.00, 95% CI 1.36–3.02), positive lymph nodes (OR = 1.75, 95% CI 1.32–2.32) and major resection (OR = 2.56, 95%CI 1.85–3.58). Receipt of blood transfusion significantly correlated with worse outcomes. The model showed good discriminative ability in both training (AUC = 0.68, 95% CI 0.66–0.72) and bootstrapping validation (C‐index = 0.67, 95% CI 0.65–0.70) cohorts. An online risk calculator of blood transfusion requirement was developed (https://catalano-giovanni.shinyapps.io/TransfusionRisk).

**Conclusions:**

Intraoperative blood transfusion was significantly associated with poor postoperative outcomes among patients undergoing surgery for ICC. The identification of patients at high risk of transfusion could improve perioperative patient care and blood resources allocation.

## 1 Introduction

Intrahepatic cholangiocarcinoma (ICC) represents approximately 15% of all primary liver tumors [1]. In addition, the incidence of ICC has been rising over the last several decades in the United States, as well as globally. Curative‐intent liver resection is the primary curative‐intent treatment option for patients with resectable, nonmetastatic ICC [2]. Despite advancements in surgical techniques, hepatic resection can still be associated with a high incidence of morbidity (22.5%) and mortality (2%–5%) [3]. In particular, peri‐operative transfusion remains more common in the setting of a liver resection than many other complex gastrointestinal operations [4]. Although blood transfusions can be critical to maintain hemodynamic stability in the setting of acute hemorrhage, as well as treat postoperative anemia, perioperative blood transfusions have been associated with poor short‐ and long‐term perioperative outcomes [5, 6, 7, 8]. Blood transfusion has been associated with increased transmission of infections, transfusion‐related acute lung injury, transfusion‐associated circulatory overload, and disease recurrence [9, 10, 11]. Informing patients about the risk of a blood transfusion is a routine part of surgical informed consent [12, 13]. As part of the informed consent process, many patients frequently inquire as to the likelihood that a transfusion will be administered in the peri‐operative period. The risk of transfusion with liver resection varies widely, however, ranging from 0% to 42% [14, 15].

To date, most predictive models of blood transfusion requirement during liver surgery have been developed based on single‐center cohorts, thus limiting the generalizability to external patient populations [14]. In addition, many studies have focused on subspecialties such as cardiac and vascular surgical procedures [16, 17], or have examined risk related to transplantation or massive transfusion [18]. Surgical resection of ICC may be particularly challenging as many patients are often asymptomatic and present with large tumors necessitating major hepatectomy [19]. While several previous studies have proposed tools to predict transfusion risk before abdominal surgery [20, 21], no preoperative risk score has been proposed to date that specifically focuses on patients with ICC undergoing hepatic resection. Therefore, the objective of the current study was to utilize an international, multi‐institutional cohort to develop a preoperative risk‐score to stratify patients relative to the likelihood of receiving an intra‐operative transfusion related to curative‐intent liver surgery for ICC. An easy‐to‐use web‐based calculator was developed and made available online to facilitate broad clinical applicability of the prediction model.

## 2 Materials and Methods

### 2.1 Study Population

Patients undergoing curative‐intent liver resection for ICC between 1990 and 2020 were identified from a multi‐institutional database of 15 tertiary institutions that comprise the International Intrahepatic Cholangiocarcinoma Study Group [22, 23, 24, 25]. Patients undergoing palliative surgery were excluded. The Institutional Review Board of each participating institution approved this study.

### 2.2 Variables and Outcomes of Interest

Demographic, clinicopathologic, and treatment characteristics of interest included age, sex, American Society of Anesthesiologist (ASA) classification, body mass index (BMI), preoperative comorbidities such as history of cirrhosis or viral hepatitis (i.e., hepatitis B [HepB] or C [HepC]), preoperative laboratory markers, including carbohydrate antigen (CA) 19‐9, total bilirubin, and hemoglobin (Hb) levels, preoperative anemia, tumor T‐ and N‐stage, receipt of transfusion and extent of resection (i.e., minor or major).

Transfusion was defined as receipt of packed red blood cells (pRBCs) intraoperatively. The grade of preoperative anemia was categorized as “severe” if preoperative Hb levels were < 10 and < 11 g/dL for females and males, respectively; “mild” anemia was defined as Hb levels < 12 and < 13 g/dL for females and males, respectively [26, 27, 28]. The definition of T‐ and N‐stage categories was based on the American Joint Committee on Cancer (AJCC) 8th edition staging manual [29]. The extent of liver resection was defined as major if three or more liver segments were resected, according to Couinaud's classification [30].

Postoperative outcomes of interest included hospital length‐of‐stay, complications within 90 days, postoperative mortality within 90 days, disease recurrence, and median overall survival (OS). OS was defined as the time interval between the date of hepatic resection and the date of death from any cause or last follow‐up.

### 2.3 Statistical Analysis and Model Development

Continuous variables were reported as median (interquartile range [IQR]) and categorical variables were reported as frequencies (proportion, %). Continuous variables were compared using the Wilcoxon rank sum test. Categorical variables were compared using the chi‐squared test or Fisher's exact test, as appropriate. Differences in survival among groups were assessed with the Kaplan–Meier method and compared with the log‐rank test. The Multiple Imputation by Chained Equations (MICE) method was utilized to impute missing data [31].

Univariable and multivariable logistic regression analyses were used to assess the association of clinicopathologic variables with blood transfusion. Variables with a *p*‐value < 0.1 in the univariable model were included in the multivariable regression analysis. Odds ratios (ORs) were presented with 95% confidence intervals (95% CI). The β‐coefficients of the statistically significant variables (*p* < 0.05) in the multivariable model were used to develop a weighted risk score. The discrimination performance of the predictive model was evaluated using the area under the curve (AUC) of the receiver operating characteristics (ROC) curve. The model was internally validated using the bootstrapping resampling method with 5000 iterations. The c‐index was calculated for the bootstrapping cohort, and the model's calibration was tested by plotting the predicted probabilities against the observed outcomes in the validation cohort. Patients with ICC undergoing liver surgery were categorized as low, medium, or high risk for intraoperative transfusion using the interquartile range values as cut‐offs. All tests were two‐sided, and a *p*‐value < 0.05 was considered statistically significant. All statistical analyses were performed using R version 4.3.2 (R Foundation for Statistical Computing, Vienna, Austria).

## 3 Results

### 3.1 Baseline Characteristics of Patient Cohort

The final cohort consisted of 1420 patients undergoing liver resection for ICC who met inclusion criteria (Table 1). Median patient age was 62 years (IQR, 53.9–70.4) and median BMI was 25.3 kg/m^2^ (IQR, 22.4–28.2). Most patients were female (*n* = 648, 45.6%) and had an ASA class ≤ 2 (*n* = 852, 60.0%). A small subset of patients had cirrhosis (*n* = 152, 11.0%), HepB (*n* = 305, 21.0%), or HepC (*n* = 52%, 3.7%). Median CA 19‐9 was 116.1 U/mL (IQR, 25.3–487.5), median total bilirubin was 0.7 mg/dL (IQR, 0.5–1.1), and median Hb was 12.8 g/dL (IQR, 11.9–14.0). Mild and severe preoperative anemia was present in 377 (27.0%) and 150 (11.0%) patients, respectively. Approximately one in two patients had T3 and T4 stage disease (*n* = 656, 46.6%), while 422 (30.0%) patients had nodal involvement (N1). A total of 862 (61.0%) patients underwent major hepatic resection.

**Table 1:** Characteristics of the study cohort.

Variable	Overall *N* = 1420	Intraoperative transfusion		*p*‐value^a^
		Not transfused *N* = 1120	Transfused *N* = 300	
Age (years)	62.0 (53.9, 70.4)	61.0 (53.0, 70.0)	62.0 (55.0, 71.0)	0.11
Female sex	648 (46.0%)	497 (44.0%)	151 (50.0%)	0.066
BMI (kg/m^2^)	25.3 (22.4, 28.2)	25.3 (22.4, 28.1)	25.3 (22.4, 28.6)	0.89
ASA‐PS > 2	568 (40.0%)	429 (38.0%)	139 (46.0%)	**0.012**
Cirrhosis	152 (11.0%)	131 (12.0%)	21 (7.0%)	**0.019**
Hepatitis B	305 (21.0%)	257 (23.0%)	48 (16.0%)	**0.009**
Hepatitis C	52 (3.7%)	37 (3.3%)	15 (5.0%)	0.16
CA 19‐9 (U/mL)	116.1 (25.3, 487.5)	100.5 (21.7, 487.0)	180.5 (39.8, 548.5)	**0.011**
Total bilirubin (mg/dL)	0.70 (0.5, 1.1)	0.7 (0.5, 1.0)	0.8 (0.5, 1.1)	**0.016**
Preoperative hemoglobin (g/dL)	12.8 (11.9, 14.0)	12.9 (12.1, 14.1)	12.4 (11.5, 13.7)	**< 0.001**
Anemia class				**0.001**
Nonanemic	893 (63.0%)	726 (65.0%)	167 (56.0%)	
Mild	377 (27.0%)	291 (26.0%)	86 (29.0%)	
Severe	150 (11.0%)	103 (9.2%)	47 (16.0%)	
AJCC T stage				**< 0.001**
T1	307 (22.0%)	273 (24.0%)	34 (11.0%)	
T2	457 (32.0%)	370 (33.0%)	87 (29.0%)	
T3	647 (46.0%)	474 (42.0%)	173 (58.0%)	
T4	9 (0.6%)	3 (0.3%)	6 (2.0%)	
AJCC N1 stage	422 (30.0%)	297 (27.0%)	125 (42.0%)	**< 0.001**
Major resection	862 (61.0%)	624 (56.0%)	238 (79.0%)	**< 0.001**

*Note:* Continuous variables: median (IQR)/categorical variables: *n* (%).

Abbreviations: AJCC, American Joint Committee on Cancer; ASA‐PS, American Society of Anesthesiology Physical Status; BMI, body mass index; CA 19‐9, carbohydrate antigen 19‐9.

a Bold values represent statistical significance.

### 3.2 Association of Clinicopathologic Characteristics With Blood Transfusion

Among patients undergoing curative‐intent liver resection for ICC, 300 individuals received an intraoperative blood transfusion (21.1%), whereas 1120 (78.9%) patients were not transfused. Patients who received a blood transfusion had more frequently an ASA score > 2 (*n* = 139, 46.0% vs. *n* = 429, 38.8%; *p* = 0.012), higher preoperative CA 19‐9 levels (180.5 [IQR, 39.8–548.5] vs. 100.5 [IQR, 21.7–487.0]; *p* = 0.011), AJCC T3 or T4 stage (*n* = 179, 60.0% vs. *n* = 477, 42.3%; *p* < 0.001) and metastatic lymph nodes (*n* = 125, 42.0% vs. *n* = 297, 27.0%; *p* < 0.001). Furthermore, patients who received intraoperative blood transfusion were less likely to have cirrhosis (*n* = 21, 7.0% vs. *n* = 131, 12.0%; *p* = 0.019) and HepB (*n* = 48, 16.0% vs. *n* = 257, 23.0%). In addition, patients who were transfused intraoperatively had lower preoperative Hb levels (12.4 [IQR, 11.5–13.7] vs. 12.9 [IQR, 12.1–14.1]; *p* < 0.001) and were more likely to have mild (*n* = 86, 29.0% vs. *n* = 291, 26.0%) or severe (*n* = 47, 16.0% vs. *n* = 103, 9.2%) anemia versus patients who were not transfused (*p* < 0.001) (Table 1). Major liver resection was more common among individuals who received an intraoperative blood transfusion (*n* = 238, 79.0% vs. *n* = 624, 56.0%; *p* < 0.001).

### 3.3 Association of Intraoperative Blood Transfusion With Postoperative Outcomes

**Figure 1:**
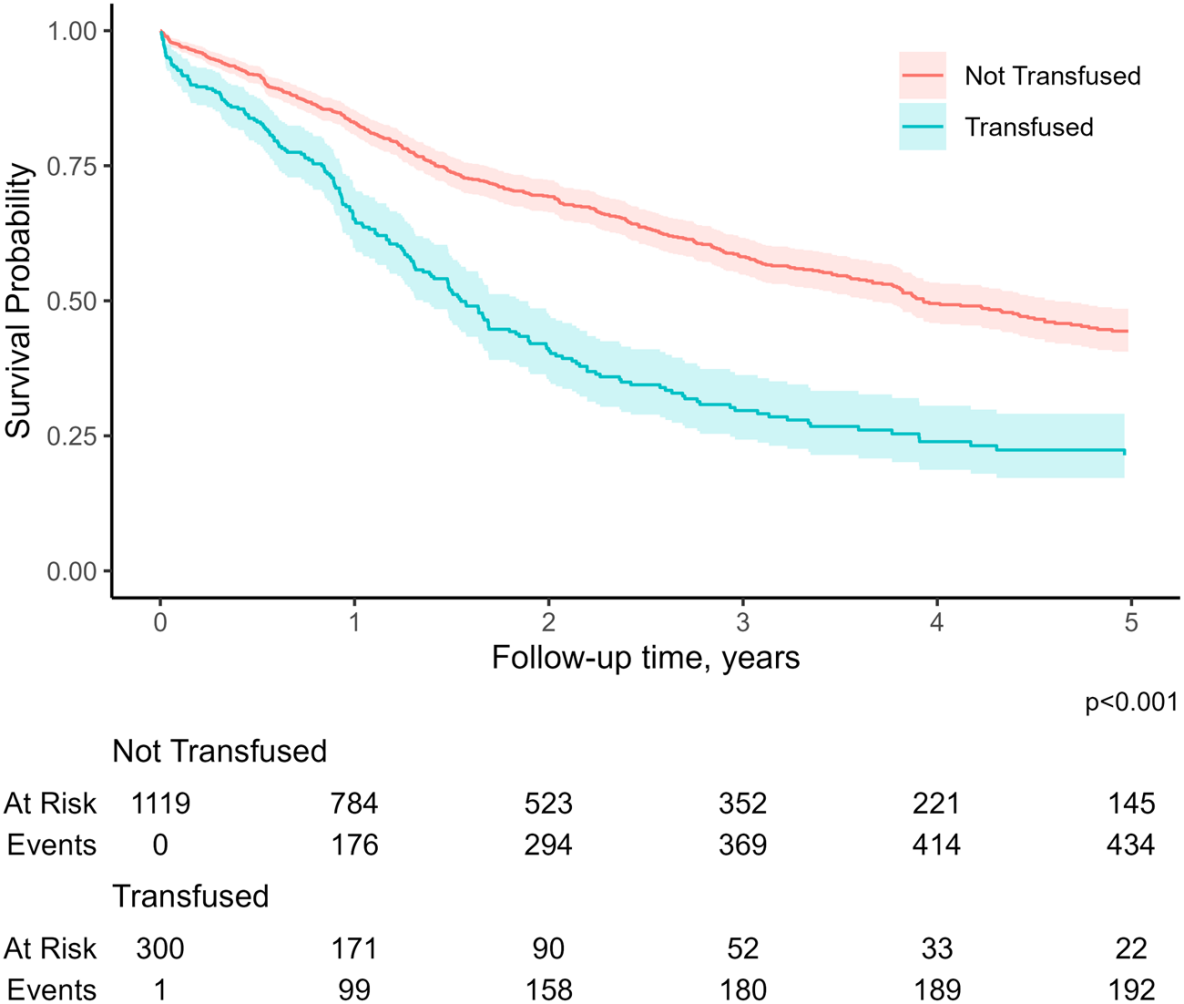
Kaplan–Meier curves of overall survival in patients who did and did not receive blood transfusions (log‐rank test: *p* < 0.001).

Patients who received intraoperative transfusion at the time of liver resection were more likely to have an extended length of stay (15 days [IQR, 10–27] vs. 11 days [IQR, 7–16]) and develop complications (*n* = 156, 52.0% vs. *n* = 313, 28.0%); patients who received an intraoperative transfusion also had a higher likelihood of postoperative mortality (*n* = 25, 8.3% vs. *n* = 15, 1.3%). Disease recurrence (*n* = 201, 67.0% vs. *n* = 662, 59.0%), as well as a worse median survival (15.2 months [IQR, 7.3–30.2] vs. 35.0 months [IQR, 14.2–59.6]) were more common among patients who received a blood transfusion compared with nontransfused patients (all *p* < 0.05, Table 2). In turn, receipt of intraoperative blood transfusion was associated with worse overall survival (*p* < 0.001, Figure 1).

**Table 2:** Postoperative outcomes.

Variable	Overall *N* = 1420	Intraoperative transfusion		*p*‐value^a^
		Not transfused *N* = 1120	Transfused *N* = 300	
Length of stay (days)	12.00 (7.00, 17.25)	11.00 (7.00, 16.00)	15.00 (10.00, 27.00)	**< 0.001**
Complication	469 (33%)	313 (28%)	156 (52%)	**< 0.001**
Postoperative mortality	40 (2.8%)	15 (1.3%)	25 (8.3%)	**< 0.001**
Recurrence	863 (61%)	662 (59%)	201 (67%)	**0.013**
OS (months)	28.67 (11.84, 59.57)	35.03 (14.23, 59.57)	15.18 (7.33, 30.22)	**< 0.001**

*Note:* Continuous variables: median (IQR)/categorical variables: *n* (%).

Abbreviation: OS, overall survival.

a Bold values represent statistical significance.

### 3.4 Independent Predictors of Intraoperative Transfusion and Risk Score Development

**Figure 2:**
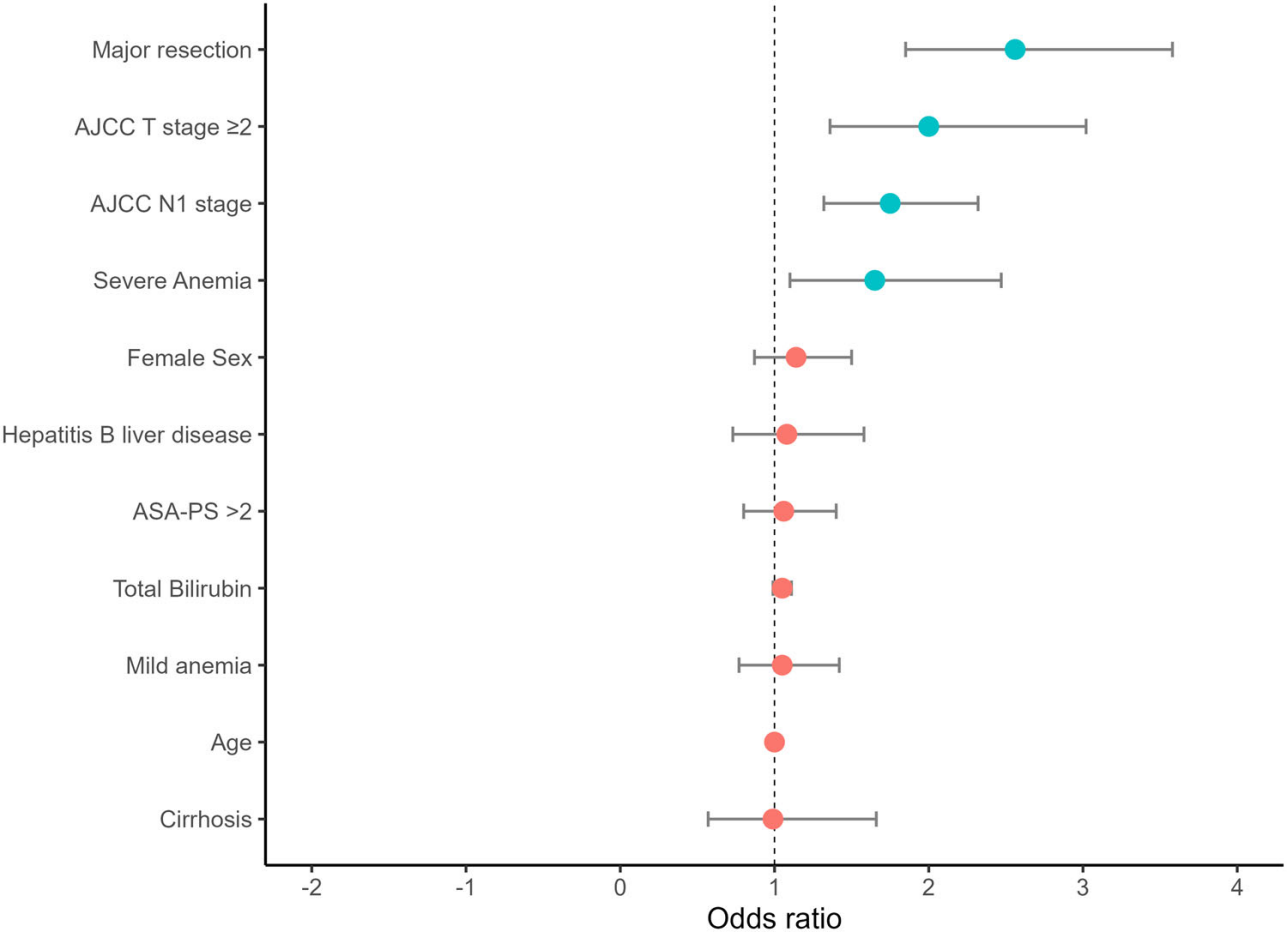
Forest plot of multivariable logistic regression analysis of the transfusion‐risk model. Variables in cyan represent statistical significance (*p* < 0.05). ASA‐PS, American Society of Anesthesiology physical status.

On multivariable analysis, after adjusting for relevant clinicopathologic factors, severe preoperative anemia (OR 1.65, 95% CI 1.10–2.47), ≥ T2 disease (OR = 2.00, 95% CI 1.36–3.02), presence of lymph node metastasis (OR 1.75, 95% CI 1.32–2.32), as well as major liver resection (OR 2.56, 95% CI 1.85–3.58) were independently associated with receipt of blood transfusion among patients undergoing curative‐intent liver surgery for ICC (Table 3, Figure 2). A risk score was subsequently developed that incorporated the independent predictors of intraoperative transfusion requirement, based on the following formula:

$$P=1/\left(1+e^{-(-2.971+0.5\;\text{if}\;\text{severe}\;\text{anemia}+0.05\;\text{if}\;\text{mild}\;\text{anemia}+0.94\;\text{if}\;\text{major}\;\text{resection}+0.69\;\text{if}\;T2\;\text{stage}\;\text{or}\;\text{higher}+0.56\;\text{if}\;N1\;\text{stage})}\right).$$

**Table 3:** Logistic regression analysis of factors associated with intraoperative blood transfusion.

Variables	Univariable			Multivariable		
	OR	95% CI	*p*‐value^a^	OR	95% CI	*p*‐value^a^
Age	1.01	1.00, 1.02	**0.091**	1.00	0.99, 1.01	0.99
Female sex	1.27	0.98, 1.64	**0.066**	1.14	0.87, 1.50	0.33
BMI	1.00	0.98, 1.03	0.94			
ASA‐PS > 2	1.39	1.07, 1.80	**0.012**	1.06	0.80, 1.40	0.68
Cirrhosis	0.57	0.34, 0.90	**0.021**	0.99	0.57, 1.66	0.98
Hepatitis B	0.64	0.45, 0.89	**0.010**	1.08	0.73, 1.58	0.69
Hepatitis C	1.54	0.81, 2.79	0.17			
CA 19‐9	1.00	1.00, 1.00	0.47			
Total bilirubin	1.08	1.02, 1.14	**0.004**	1.05	0.99, 1.11	0.076
Anemia class						
Nonanemic	ref	ref		ref	ref	
Mild	1.28	0.96, 1.72	**0.094**	1.05	0.77, 1.42	0.77
Severe	1.98	1.34, 2.90	**< 0.001**	1.65	1.10, 2.47	**0.014**
AJCC T stage ≥ 2	2.52	1.74, 3.75	**< 0.001**	2.00	1.36, 3.02	**< 0.001**
AJCC N1 stage	1.98	1.52, 2.58	**< 0.001**	1.75	1.32, 2.32	**< 0.001**
Major resection	3.05	2.27, 4.16	**< 0.001**	2.56	1.85, 3.58	**< 0.001**

Abbreviations: ASA‐PS, American Society of Anesthesiology Physical Status; BMI, body mass index; CA 19‐9, carbohydrate antigen 19‐9; CI, confidence interval; OR, odds ratio.

a Bold values represent statistical significance.

**Figure 3:**
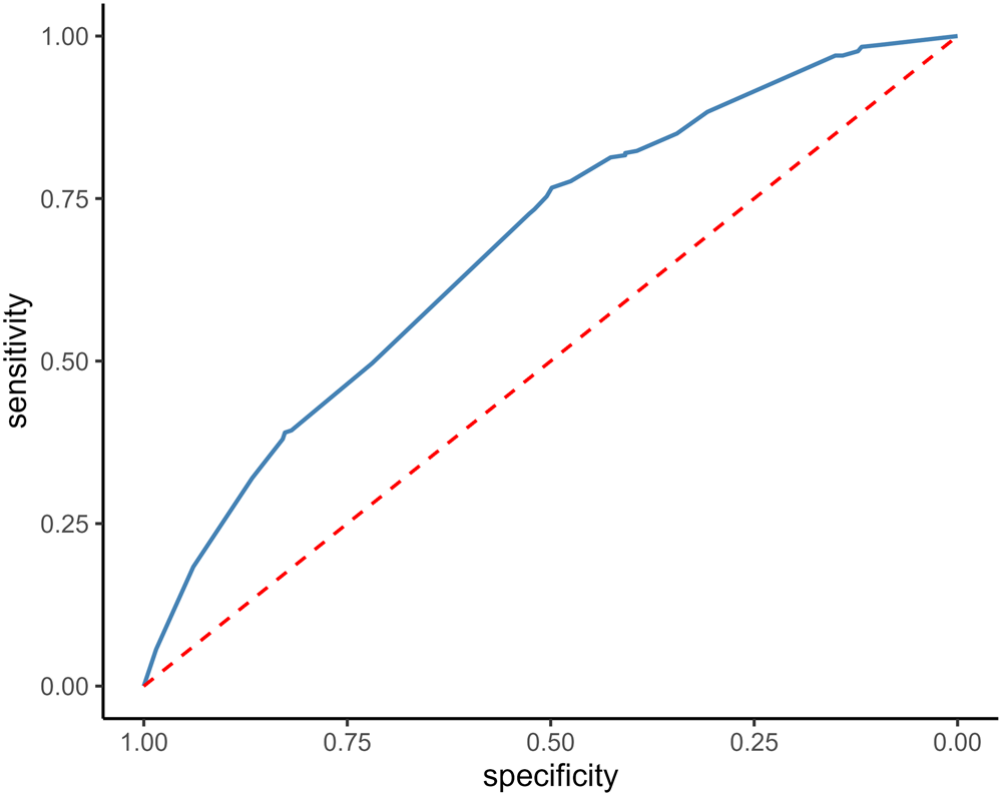
Transfusion risk score ROC curve (AUC = 0.68, 95% CI 0.66–0.72). AUC, area under the curve; ROC, receiver operating characteristics.

**Figure 4:**
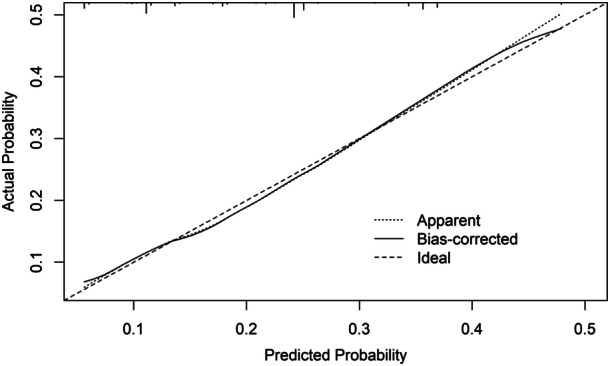
Calibration plot of the transfusion risk model after internal validation (bootstrapping, 5000 repetitions).

**Figure 5:**
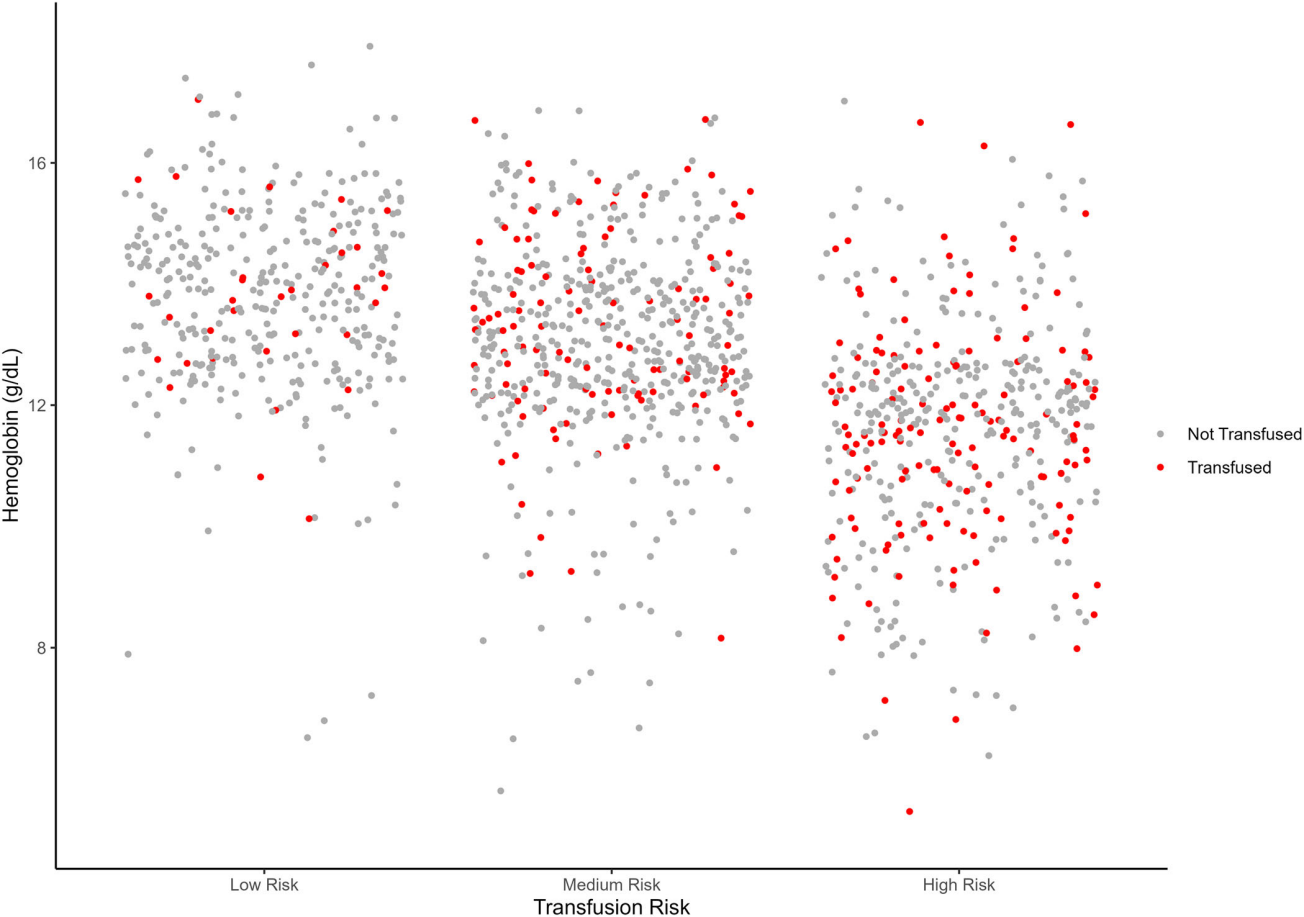
Risk of intraoperative transfusion according to preoperative hemoglobin levels.

**Figure 6:**
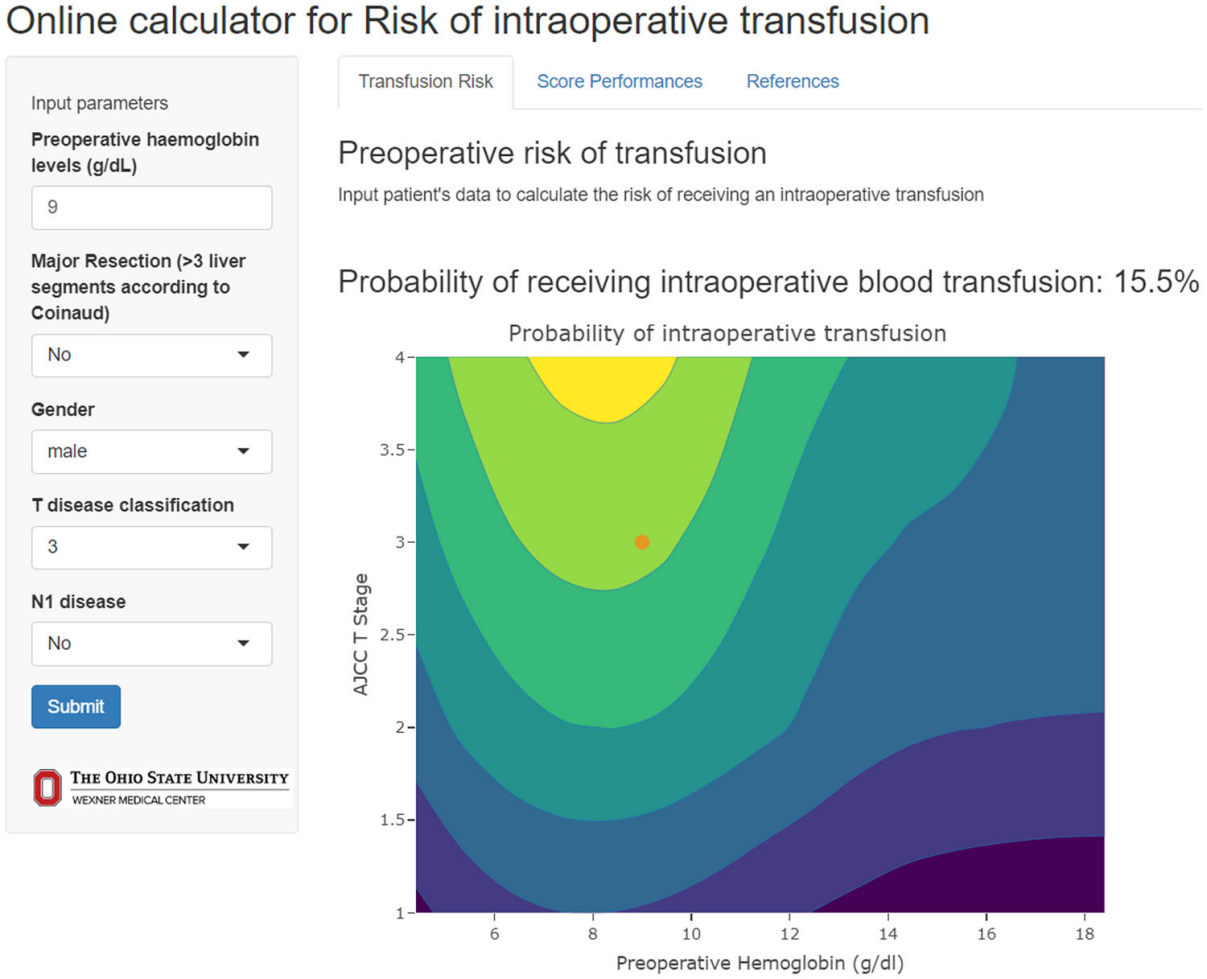
User interface of the online calculator and the interactive contour plot.

The risk score had an AUC of 0.68 (95% CI 0.66–0.72) in the training cohort (Figure 3) and its predictive accuracy was confirmed in the internal validation cohort with bootstrapping resamples (AUC 0.67 [95% CI 0.65–0.70]). The calibration plot indicated that the estimated probability and the observed frequency were in good agreement (Figure 4). Using the risk score, patients with ICC were categorized as being low, medium, or high risk of receiving intraoperative blood transfusion (Figure 5). The relationship between preoperative hemoglobin levels, T classification, and the probability of intraoperative blood transfusion was depicted with an interactive contour plot; in addition, an easy‐to‐use calculator was developed to predict the need for intraoperative blood transfusion (available at: https://catalano-giovanni.shinyapps.io/TransfusionRisk) (Figure 6).

## 4 Discussion

ICC is a highly aggressive tumor with an increasing incidence worldwide [1]. Curative‐intent hepatic resection is the main treatment for resectable, nonmetastatic ICC [2]. Despite substantial advancements in surgical techniques and perioperative strategies in recent years, prognosis even after hepatic resection remains poor with a median overall survival of 15–31 months [32]. Intraoperative bleeding is a common complication associated with liver surgery [3]. In turn, utilization of blood transfusion may be higher among patients with ICC who often present with large tumors in need of a major hepatic resection [15]. Notably, receipt of blood transfusion during hepatic surgery has been associated with transfusion‐related complications, high mortality, and worse cancer‐specific outcomes [33, 34, 35, 36]. Meanwhile, there is an ongoing blood product shortage worldwide, highlighting the need for improved strategies of resource allocation and conservation [37]. The current study was important because we used an international, multi‐institutional cohort to identify independent predictors of intraoperative blood transfusion among patients undergoing surgery for ICC. Of note, roughly one in five patients undergoing curative‐intent liver resection for ICC received an intraoperative blood transfusion. The likelihood of a blood transfusion was associated with worse patient (i.e., anemia), tumor (i.e., T and N classification), as well as procedure (i.e., major resection) characteristics. In addition, receipt of an intraoperative transfusion was correlated with worse short‐ and long‐term outcomes. A preoperative risk‐score to estimate the probability of blood transfusion among patients undergoing curative‐intent liver resection for ICC was developed and made available as a practical, online risk calculator.

Blood transfusion during surgery has been associated with adverse oncologic outcomes in various malignancies including gastric, pancreatic, hepatocellular, and lung cancer [36]. One proposed mechanism involves the immunomodulatory role of blood transfusion, which can decrease inteleukin‐2 levels and cytotoxic T‐cell activity, while elevating levels of immunosuppressive prostaglandins [36, 38, 39, 40]. Via these effects on the immune system, blood transfusion may mitigate tumor immune response and promote systemic inflammatory response; thus leading to increased morbidity and disease recurrence. Postlewait et al. reported that transfusion was associated with major complications and 90‐day readmission [36]. In a different study, Kim et al. demonstrated that transfusion within 72 hours after surgery was an independent predictor of unplanned readmissions [41]. In the current study, patients who received intraoperative transfusion at the time of liver resection for ICC were more likely to have an extended length of stay (15 days [IQR, 10–27] vs. 11 days [IQR, 7–16]) and develop complications (*n* = 156, 52.0% vs. *n* = 313, 28.0%); patients who received an intraoperative transfusion also had a higher likelihood of postoperative mortality (*n* = 25, 8.3% vs. *n* = 15, 1.3%). Other data have suggested that peri‐operative blood transfusions can lead to worse oncologic outcomes [5]. In fact, peri‐operative blood transfusion has been associated with a 5‐year OS almost 2.5 times lower than in patients who did not receive a transfusion [6, 7, 8]. In the current study, receipt of an intra‐operative transfusion at the time of liver resection for ICC was associated with increased disease recurrence, as well as worse survival.

To date, few predictive models have been developed to assess the potential need for blood transfusion among patients undergoing hepatic resection [42, 43, 44]. Additionally, most existing models were developed using cohorts from a single institution, thus limiting their validity among more diverse patient populations [14]. For example, Lemke et al. evaluated three, single‐center transfusion risk scores, in addition to developing and validating a simplified three‐point risk score [44]. The three‐point risk score incorporated preoperative anemia, primary liver cancer diagnosis, and major resection as predictive factors. This score had an AUC of 0.66 (95% CI 0.63–0.69), which was comparable to the reported accuracy of other published prediction tools (Cockbain et al. [45]: 0.66, 95% CI 0.63–0.69; Sima et al. [46]: 0.66, 95% CI 0.63–0.70; Pulitanò et al. [43]: 0.68, 95% CI 0.64–0.71). In a different, single‐institution, study of patients undergoing hepatic resection for HepB‐related HCC, Wang et al. developed a risk score for transfusion based on extent of resection, extrahepatic procedures, hemoglobin and platelet levels (AUC 0.74) [42]. In the current study, independent predictors of intraoperative blood transfusion related to ICC included extent of preoperative anemia, AJCC T and N disease, as well as extent of resection. Utilizing these factors, a risk score to predict the likelihood of an intraoperative transfusion was developed. Of note, the score performed comparably to other reported prediction tools in both the training (AUC 0.68) and validation (AUC 0.67) cohorts with good calibration [43, 45, 46]. The risk score was able to stratify patients into three groups; low, medium, and high risk for intraoperative blood transfusion requirement.

The availability of a tool to predict the probability of blood transfusion in the preoperative setting has important clinical implications. A tool to guide the likelihood of transfusion may help inform which patients may benefit from referral to the blood management program [47]. Preoperative blood ordering involves screening for red cell antibodies and/or crossmatch with specific units of packed red blood cells in anticipation of a possible transfusion [48]. Excessive crossmatch of blood before the operation can overburden blood bank personnel, as well as lead to outdating of blood, depletion of blood bank resources, and increased hospital costs [49]. Our group has previous reported that more than one in four patients receive crossmatch orders that exceeded national guidelines. In addition, there is a marked provider variation among surgeons associated with ordering of blood for crossmatch in the preoperative setting [48]. More accurate prediction of transfusion needs in the preoperative setting may reduce excess crossmatch ordering before surgery. In addition, a better understanding of the likelihood of an intraoperative transfusion may inform the need of autologous blood donation, as well as making cell‐saver available for autotransfusion in the operating room [50, 51]. Furthermore, the identification of patients at higher risk for blood transfusion could aid in better allocating blood product resources, avoid blood product waste, and, thus, improve cost‐effectiveness. The availability of the tool in an easy‐to‐use web calculator can facilitate its clinical application (https://catalano-giovanni.shinyapps.io/TransfusionRisk).

The present study should be interpreted in light of several limitations. Due to the retrospective design of the study, selection and reporting biases were possible. Although the current analysis considered many factors related to transfusion risk, other factors such as surgical technique, the use of blood flow management strategies, and the experience of the Anesthesia team may have influenced the results. In fact, even though the multi‐institutional nature of the cohort was a strength, perioperative clinical and surgical management, as well as intraoperative transfusion practices may have varied among different institutions. Further validation of the predictive risk score in external cohorts is therefore necessary.

## 5 Conclusions

In conclusion, one in five patients undergoing resection of ICC received an intraoperative blood transfusion. Blood transfusion was associated with worse clinicopathologic characteristics and poor postoperative outcomes. A preoperative risk score to predict the likelihood of a blood transfusion during surgery for ICC was developed based on patient, tumor, and procedure‐related factors. The tool had good accuracy and was made available via an on‐line calculator to assist clinicians. The preoperative risk stratification of patients relative to likelihood of a transfusion can assist informed consent, as well as help with peri‐operative planning relative to curative‐intent liver resection for ICC.

## Conflicts of Interest

The authors declare no conflicts of interest.

## Synopsis

Patients undergoing liver resection surgery for intrahepatic cholangiocarcinoma have a high risk of perioperative bleeding. Intraoperative blood transfusions are associated with worse postoperative outcomes. Our study aimed to develop a preoperative risk calculator to identify patients at higher risk for intraoperative transfusion, aiding in better blood products allocation.

## Data Availability

The data that support the findings of this study are available on request from the corresponding author. The data are not publicly available due to privacy or ethical restrictions.

## References

[1] A. K. Singal , J.‐N. Vauthey , J. J. Grady , and J. R. Stroehlein , “Intra‐Hepatic Cholangiocarcinoma—Frequency and Demographic Patterns: Thirty‐Year Data From the M.D. Anderson Cancer Center,” Journal of Cancer Research and Clinical Oncology 137 (2011): 1071–1078. 21207060 10.1007/s00432-010-0971-z PMC11827973

[2] A. B. Benson , M. I. D'Angelica , D. E. Abbott , et al., “Hepatobiliary Cancers, Version 2.2021, NCCN Clinical Practice Guidelines in Oncology,” Journal of the National Comprehensive Cancer Network 19 (2021): 541–565. 34030131 10.6004/jnccn.2021.0022

[3] P. J. Kneuertz , H. A. Pitt , K. Y. Bilimoria , et al., “Risk of Morbidity and Mortality Following Hepato‐Pancreato‐Biliary Surgery,” Journal of Gastrointestinal Surgery 16 (2012): 1727–1735. 22760965 10.1007/s11605-012-1938-y

[4] A. J. Kansagra and M. S. Stefan , “Preoperative Anemia,” Anesthesiology Clinics 34 (2016): 127–141. 26927743 10.1016/j.anclin.2015.10.011

[5] M. N. Mavros , L. Xu , H. Maqsood , et al., “Perioperative Blood Transfusion and the Prognosis of Pancreatic Cancer Surgery: Systematic Review and Meta‐Analysis,” Annals of Surgical Oncology 22 (2015): 4382–4391. 26293837 10.1245/s10434-015-4823-6

[6] A. G. Acheson , M. J. Brookes , and D. R. Spahn , “Effects of Allogeneic Red Blood Cell Transfusions on Clinical Outcomes in Patients Undergoing Colorectal Cancer Surgery: A Systematic Review and Meta‐Analysis,” Annals of Surgery 256 (2012): 235–244. 22791100 10.1097/SLA.0b013e31825b35d5

[7] A. Amato and M. Pescatori , “Perioperative Blood Transfusions and Recurrence of Colorectal Cancer,” Cochrane Database Systematic Review 2011, no. 1 (2006): CD005033, 10.1002/14651858.CD005033.pub2. PMC6486137 16437512

[8] C. Sun , Y. Wang , H. S. Yao , and Z. Q. Hu , “Allogeneic Blood Transfusion and the Prognosis of Gastric Cancer Patients: Systematic Review and Meta‐Analysis,” International Journal of Surgery 13 (2015): 102–110. 25486261 10.1016/j.ijsu.2014.11.044

[9] N. Amini , G. Spolverato , Y. Kim , and T. M. Pawlik , “Trends in Hospital Volume and Failure to Rescue for Pancreatic Surgery,” Journal of Gastrointestinal Surgery 19 (2015): 1581–1592. 25794484 10.1007/s11605-015-2800-9

[10] S. A. Ahmad , M. J. Edwards , J. M. Sutton , et al., “Factors Influencing Readmission After Pancreaticoduodenectomy: A Multi‐Institutional Study of 1302 Patients,” Annals of Surgery 256 (2012): 529–537. 22868373 10.1097/SLA.0b013e318265ef0b

[11] C. N. Clarke , J. J. Sussman , D. E. Abbott , and S. A. Ahmad , “Factors Affecting Readmission After Pancreaticoduodenectomy,” Advances in Surgery 47 (2013): 99–110. 24298846 10.1016/j.yasu.2013.02.006

[12] M. F. Murphy , A. Harris , J. Neuberger , et al., “Consent for Blood Transfusion: Summary of Recommendations From the Advisory Committee for the Safety of Blood, Tissues and Organs (SaBTO),” Clinical Medicine 21 (2021): 201–203. 34001570 10.7861/clinmed.2020-1035 PMC8140698

[13] National Institute for Health and Care Excellence (NICE) . NICE Guideline, No. 24. 20, Patient Information. Blood Transfusion. (London: National Clinical Guideline Centre (UK), 2015), https://www.ncbi.nlm.nih.gov/books/NBK338774/. 26632625

[14] J. L. J. Chin , J. C. Allen , Y.‐X. Koh , et al., “Poor Utility of Current Nomograms Assessing the Risk of Intraoperative Blood Transfusion in Patients Undergoing Liver Resection for Hepatocellular Carcinoma and Proposal of a New Model,” Surgery 172 (2022): 1442–1447. 36038372 10.1016/j.surg.2022.06.007

[15] C. W. Kimbrough , S. C. Agle , C. R. Scoggins , et al., “Factors Predictive of Readmission After Hepatic Resection for Hepatocellular Carcinoma,” Surgery 156 (2014): 1039–1048. 25086792 10.1016/j.surg.2014.06.057

[16] C. B. C. Cunha , T. A. Lima , D. L. M. Ferraz , et al., “Predicting the Need for Blood Transfusions in Cardiac Surgery: A Comparison between Machine Learning Algorithms and Established Risk Scores in the Brazilian Population,” Brazilian Journal of Cardiovascular Surgery 39 (2024): e20230212. 38426717 10.21470/1678-9741-2023-0212 PMC10903744

[17] J. Leff , C. A. Romano , S. Gilbert , and S. Nair , “Validation Study of the Transfusion Risk and Clinical Knowledge (TRACK) Tool in Cardiac Surgery Patients: A Retrospective Analysis,” Journal of Cardiothoracic and Vascular Anesthesia 33 (2019): 2669–2675. 31227377 10.1053/j.jvca.2019.05.040

[18] S. M. Lee , G. Lee , T. K. Kim , et al., “Development and Validation of a Prediction Model for Need for Massive Transfusion During Surgery Using Intraoperative Hemodynamic Monitoring Data,” JAMA Network Open 5 (2022): e2246637. 36515949 10.1001/jamanetworkopen.2022.46637 PMC9856486

[19] K. Wang , H. Zhang , Y. Xia , J. Liu , and F. Shen , “Surgical Options for Intrahepatic Cholangiocarcinoma,” HepatoBiliary Surgery and Nutrition 6 (2017): 79–90. 28503555 10.21037/hbsn.2017.01.06 PMC5411277

[20] Y. Kim , F. Bagante , F. Gani , et al., “Nomogram to Predict Perioperative Blood Transfusion for Hepatopancreaticobiliary and Colorectal Surgery,” British Journal of Surgery 103 (2016): 1173–1183. 27222214 10.1002/bjs.10164

[21] K. Merath , J. M. Hyer , R. Mehta , et al., “Use of Machine Learning for Prediction of Patient Risk of Postoperative Complications After Liver, Pancreatic, and Colorectal Surgery,” Journal of Gastrointestinal Surgery 24 (2020): 1843–1851. 31385172 10.1007/s11605-019-04338-2

[22] L. Alaimo , Y. Endo , G. Catalano , et al., “Benchmarks in Liver Resection for Intrahepatic Cholangiocarcinoma,” Annals of Surgical Oncology 31 (2024): 3043–3052, 10.1245/s10434-023-14880-8. 38214817 PMC10997542

[23] D. I. Tsilimigras , D. Moris , R. Mehta , et al., “The Systemic Immune‐Inflammation Index Predicts Prognosis in Intrahepatic Cholangiocarcinoma: An International Multi‐Institutional Analysis,” HPB 22 (2020): 1667–1674. 32265108 10.1016/j.hpb.2020.03.011

[24] D. I. Tsilimigras , R. Mehta , L. Aldrighetti , et al., “Development and Validation of a Laboratory Risk Score (LabScore) to Predict Outcomes after Resection for Intrahepatic Cholangiocarcinoma,” Journal of the American College of Surgeons 230 (2020): 381–391.e2. 32014569 10.1016/j.jamcollsurg.2019.12.025

[25] D. I. Tsilimigras , J. M. Hyer , D. Moris , et al., “Prognostic Utility of Albumin‐Bilirubin Grade for Short‐ and Long‐Term Outcomes Following Hepatic Resection for Intrahepatic Cholangiocarcinoma: A Multi‐Institutional Analysis of 706 Patients,” Journal of Surgical Oncology 120 (2019): 206–213. 31025380 10.1002/jso.25486

[26] M. Domenica Cappellini and I. Motta , “Anemia in Clinical Practice—Definition and Classification: Does Hemoglobin Change With Aging?,” Seminars in Hematology 52 (2015): 261–269. 26404438 10.1053/j.seminhematol.2015.07.006

[27] H. Y. Kwon , B. R. Kim , and Y. W. Kim , “Association of Preoperative Anemia and Perioperative Allogenic Red Blood Cell Transfusion With Oncologic Outcomes in Patients With Nonmetastatic Colorectal Cancer,” Current Oncology 26 (2019): 357–366. 10.3747/co.26.4983 PMC6588057 31285680

[28] T. W. Kim , H. J. Park , M. J. Chang , et al., “Effect of Severity and Cause of Preoperative Anemia on the Transfusion Rate After Total Knee Arthroplasty,” Scientific Reports 12 (2022): 4083. 35260783 10.1038/s41598-022-08137-9 PMC8904493

[29] M. B. Amin , F. L. Greene , S. B. Edge , et al., “The Eighth Edition ajcc Cancer Staging Manual: Continuing to Build a Bridge From a Population‐Based to a More ‘Personalized’ Approach to Cancer Staging,” CA: A Cancer Journal for Clinicians 67 (2017): 93–99. 28094848 10.3322/caac.21388

[30] Y. Y. Pang and S. M. Strasberg , “The Brisbane 2000 Terminology of Liver Anatomy and Resections,” HPB 4 (2002): 99–100. 18332933 10.1080/136518202760378489 PMC2020531

[31] S. Van Buuren , H. C. Boshuizen , and D. L. Knook , “Multiple Imputation of Missing Blood Pressure Covariates in Survival Analysis,” Statistics in Medicine 18 (1999): 681–694. 10204197 10.1002/(sici)1097-0258(19990330)18:6<681::aid-sim71>3.0.co;2-r

[32] M. N. Mavros , K. P. Economopoulos , V. G. Alexiou , and T. M. Pawlik , “Treatment and Prognosis for Patients With Intrahepatic Cholangiocarcinoma: Systematic Review and Meta‐Analysis,” JAMA Surgery 149 (2014): 565. 24718873 10.1001/jamasurg.2013.5137

[33] T. A. Aloia , B. N. Fahy , C. P. Fischer , et al., “Predicting Poor Outcome Following Hepatectomy: Analysis of 2313 Hepatectomies in the NSQIP Database,” HPB 11 (2009): 510–515. 19816616 10.1111/j.1477-2574.2009.00095.x PMC2756639

[34] Y. Makino , A. Yamanoi , T. Kimoto , O. N. El‐Assal , H. Kohno , and N. Nagasue , “The Influence of Perioperative Blood Transfusion on Intrahepatic Recurrence After Curative Resection of Hepatocellular Carcinoma,” American Journal of Gastroenterology 95 (2000): 1294–1300. 10811342 10.1111/j.1572-0241.2000.02028.x

[35] A. Ejaz , G. Spolverato , Y. Kim , et al., “Impact of Blood Transfusions and Transfusion Practices on Long‐Term Outcome Following Hepatopancreaticobiliary Surgery,” Journal of Gastrointestinal Surgery 19 (2015): 887–896. 25707813 10.1007/s11605-015-2776-5

[36] L. M. Postlewait , M. H. Squires , D. A. Kooby , et al., “The Relationship of Blood Transfusion With Peri‐Operative and Long‐Term Outcomes After Major Hepatectomy for Metastatic Colorectal Cancer: A Multi‐Institutional Study of 456 Patients,” HPB 18 (2016): 192–199. 26902139 10.1016/j.hpb.2015.08.003 PMC4814612

[37] N. Roberts , S. James , M. Delaney , and C. Fitzmaurice , “The Global Need and Availability of Blood Products: A Modelling Study,” The Lancet Haematology 6 (2019): e606–e615. 31631023 10.1016/S2352-3026(19)30200-5

[38] C. Miki , J. Hiro , E. Ojima , Y. Inoue , Y. Mohri , and M. Kusunoki , “Perioperative Allogeneic Blood Transfusion, the Related Cytokine Response and Long‐Term Survival After Potentially Curative Resection of Colorectal Cancer,” Clinical Oncology 18 (2006): 60–66. 16477921 10.1016/j.clon.2005.08.004

[39] M. Ghio , P. Contini , C. Mazzei , et al., “In Vitro Immunosuppressive Activity of Soluble Hla Class I and Fas Ligand Molecules: Do They Play a Role in Autologous Blood Transfusion?,” Transfusion 41 (2001): 988–996. 11493729 10.1046/j.1537-2995.2001.41080988.x

[40] J. P. Cata , H. Wang , V. Gottumukkala , J. Reuben , and D. I. Sessler , “Inflammatory Response, Immunosuppression, and Cancer Recurrence After Perioperative Blood Transfusions,” British Journal of Anaesthesia 110 (2013): 690–701. 23599512 10.1093/bja/aet068 PMC3630286

[41] S. Kim , E. C. Maynard , M. B. Shah , et al., “Risk Factors for 30‐Day Readmissions After Hepatectomy: Analysis of 2444 Patients From the ACS‐NSQIP Database,” Journal of Gastrointestinal Surgery 19 (2015): 266–271. 25451735 10.1007/s11605-014-2713-z

[42] H.‐Q. Wang , J. Yang , J.‐Y. Yang , W. T. Wang , and L. N. Yan , “Development and Validation of a Predictive Score for Perioperative Transfusion in Patients With Hepatocellular Carcinoma Undergoing Liver Resection,” Hepatobiliary & Pancreatic Diseases International 14 (2015): 394–400. 26256084 10.1016/s1499-3872(15)60362-9

[43] C. Pulitanò , M. Arru , L. Bellio , S. Rossini , G. Ferla , and L. Aldrighetti , “A Risk Score for Predicting Perioperative Blood Transfusion in Liver Surgery,” British Journal of Surgery 94 (2007): 860–865. 17380562 10.1002/bjs.5731

[44] HPB CONCEPT Team , M. Lemke , C. H. L. Law , J. Li , et al., “Three‐Point Transfusion Risk Score in Hepatectomy,” British Journal of Surgery 104 (2017): 434–442. 28079259 10.1002/bjs.10416

[45] A. J. Cockbain , T. Masudi , J. P. A. Lodge , G. J. Toogood , and K. R. Prasad , “Predictors of Blood Transfusion Requirement in Elective Liver Resection,” HPB 12 (2010): 50–55. 20495645 10.1111/j.1477-2574.2009.00126.x PMC2814404

[46] C. S. Sima , W. R. Jarnagin , Y. Fong , et al., “Predicting the Risk of Perioperative Transfusion for Patients Undergoing Elective Hepatectomy,” Annals of Surgery 250 (2009): 914–921. 19953711 10.1097/sla.0b013e3181b7fad3

[47] F. Gani , M. Cerullo , A. Ejaz , et al., “Implementation of a Blood Management Program at a Tertiary Care Hospital: Effect on Transfusion Practices and Clinical Outcomes Among Patients Undergoing Surgery,” Annals of Surgery 269 (2019): 1073–1079. 31082904 10.1097/SLA.0000000000002585

[48] A. Ejaz , S. M. Frank , G. Spolverato , M. Mavros , Y. Kim , and T. M. Pawlik , “Variation in the Use of Type and Crossmatch Blood Ordering Among Patients Undergoing Hepatic and Pancreatic Resections,” Surgery 159 (2016): 908–918. 26384235 10.1016/j.surg.2015.07.029

[49] A. Subramanian , K. Rangarajan , S. Kumar , K. Farooque , V. Sharma , and M. Misra , “Reviewing the Blood Ordering Schedule for Elective Orthopedic Surgeries at a Level One Trauma Care Center,” Journal of Emergencies, Trauma, and Shock 3 (2010): 225. 20930965 10.4103/0974-2700.66521 PMC2938486

[50] G. Niranjan , G. Asimakopoulos , A. Karagounis , G. Cockerill , M. Thompson , and V. Chandrasekaran , “Effects of Cell Saver Autologous Blood Transfusion on Blood Loss and Homologous Blood Transfusion Requirements in Patients Undergoing Cardiac Surgery On‐ Versus Off‐Cardiopulmonary Bypass: A Randomised Trial☆,” European Journal of Cardio‐Thoracic Surgery 30 (2006): 271–277. 16829083 10.1016/j.ejcts.2006.04.042

[51] R. L. Tawes , R. G. Scribner , T. B. Duval , et al., “The Cell‐Saver and Autologous Transfusion: An Underutilized Resource in Vascular Surgery,” The American Journal of Surgery 152 (1986): 105–109. 3089043 10.1016/0002-9610(86)90156-x

